# A Comparative Study of Five Mouse Models of Alzheimer's Disease: Cell Cycle Events Reveal New Insights into Neurons at Risk for Death

**DOI:** 10.4061/2011/171464

**Published:** 2011-09-08

**Authors:** Luming Li, Timmy Cheung, Jianmin Chen, Karl Herrup

**Affiliations:** Department of Cell Biology and Neuroscience, Rutgers University, B211 Nelson Labs, 604 Allison Road, Piscataway, NJ 08854-6999, USA

## Abstract

Ectopic cell cycle events (CCEs) in postmitotic neurons link the neurodegenerative process in human Alzheimer's disease (AD) with the brain phenotype of transgenic mouse models with known familial AD genes. Most reports on the mouse models use the appearance of brain amyloid pathology as a key outcome measure. In the current paper, we focus on the induction of neurodegeneration using CCEs as markers for impending neuronal loss. We compare 5 mouse models of familial AD for the appearance of CCEs in subcortical regions—deep cerebellar nuclei, amygdala, locus coeruleus, hippocampus, and dorsal raphe. We find that the models differ in their CCE involvement as well as in the appearance of phosphorylated tau and amyloid deposition, suggesting that each model represents a different disease phenotype. Comparison with the pattern of neuron death in human AD suggests that each may represent a distinctly different disease model when used in preclinical trials.

## 1. Introduction

Alzheimer's disease (AD) is a devastating late-onset neurodegenerative condition that affects many regions of the human brain. Although the most obvious disease symptoms involve the inability to form and store new memories, the neurological and psychiatric description of an individual with AD includes a wide range of symptoms such as depression, apathy, episodic behavioral outbursts, deteriorating executive functioning, and others. The biological substrates of these symptoms are only partially understood, but imaging and neuropathological studies have revealed important facets of their diverse and distributed nature. There is a clear loss of volume and pathologically visible degeneration in the brain's memory centers, which include the entorhinal cortex, hippocampus, and basal forebrain nucleus. But there are also functional and structural abnormalities found in the locus coeruleus, dorsal raphe, cingulate gyrus, amygdala and prefrontal cortex as well as other cortical and subcortical regions [1–3]. Amyloid plaques and neurofibrillary tangles are the widely accepted biochemical signatures of AD, used to confirm the clinical diagnosis upon final neuropathological examination. These plaques and tangles are found in conjunction with significant and progressive neurodegeneration affecting both synapses and cell bodies. While the appearance of the abnormal deposits is disease specific, their anatomical locations in human AD mark only a subset of the brain regions that are identified as undergoing significant atrophy during the progress of the disease.

Recent work from our laboratory and many others has explored the use of abnormal neuronal cell cycle processes as an additional pathological marker of disease [4–11]. The timing and location of neuronal cell death in AD has been intimately associated with the unscheduled appearance of events related to mitotic cell division. Both cell cycle-related proteins and evidence of DNA replication have been found in neurons that are considered “at risk” for death. It is hypothesized that, although the neurons are able to initiate a true cell cycle and replicate most if not all of their genome, they are incapable of completing the process and are believed to die [12]. Using immunohistochemical analysis, cell cycle events (CCEs) have been identified in subcortical brain regions of individuals with AD as well as those with mild cognitive impairment (MCI—considered by many to be the clinical precursor of AD) [11]. In age-matched controls and in AD brain regions where neurons are not susceptible to death, cell cycle-related protein expression is significantly lower. This has led to the hypothesis that cell cycle events represent the first step of a process that leads to neuronal cell death in AD. 

Significantly, these unexpected attempts by neurons to reenter a cell cycle provide one of the few homologies observed between mouse models of AD and the pathogenesis of the human condition. A number of different AD models have been created, most of which rely on transgenes encoding the gene for *β*-amyloid precursor protein (*APP*), presenilin-1 (*PSEN1*) or both [4]. Produced in a number of different laboratories, these models reproduce the plaque pathology of AD and lead to modest behavioral abnormalities. However, none produces the neurofibrillary tangles of AD or the severe behavioral changes that mark the end stages of the human disease. Other researchers have developed models of human tauopathies based on transgenes expressing disease causing variants of the microtubule-associated protein tau [13, 14]. Paradoxically, despite the fact that familial AD has not been significantly associated with tau (*MAPT*) mutations in humans, the tauopathy mouse models have been reasonably successful in reproducing many of the pathological characteristics of AD. With age, the brains of these animals display both tangles of hyperphosphorylated tau and progressive neurodegeneration in some cases [15]. Thus, *MAPT* models reproduce the tangles and degeneration but not the plaques, while the *APP/PS1* models reproduce the Alzheimer's plaques but not the associated tangles or neurodegeneration. From the standpoint of the plaques and tangles, therefore, the *APP/PS1* mice are the better genocopies of AD while the *MAPT* mice are somewhat better phenocopies.

We have elected to focus on the pattern of neurodegeneration in APP transgenic mice in order to expand the characterization of this group of AD models, and we have used CCEs as outcome measures. Previously, where they have been studied in depth, the appearance of CCEs in many human disease models show an age-dependent increase in prevalence that often closely mimics the pattern of neuronal cell death in the human disease. For example, there is a significant correlation between the regional pattern of cell loss in human ataxia-telangiectasia and its mouse model [16]. The same is true for amyotrophic lateral sclerosis [17]. For human AD, the temporal and spatial appearance of CCEs in the R1.40 AD mouse model [18–20] accurately recapitulate the anatomical progression of cell death in the human. In the current study, we expand the use of cell cycle markers as a benchmark of neuronal distress in the mouse. Our goal was to determine the phenotypic variability among the various mouse models of AD and to learn whether the similarities and differences observed are informative as to their relative fidelity to the human disease. We were particularly interested in exploring the involvement of the subcortical areas affected in AD since these regions typically receive less attention yet are likely to contribute significantly to the symptoms of AD. We focused our efforts on five of the many models available. We describe a pattern of selective neuronal vulnerability similar to human AD which is recapitulated in some, but not all of the five.

## 2. Material and Methods

### 2.1. Transgenic Mouse Models of AD

Five transgenic mouse models of familial AD were used in the current studies. In each model, amyloid plaques induced by APP develop at different ages. Detailed information about the mouse strains is summarized in Table 1. Most animals (R1.40 B6.129-Tg(APPSw)40Btla/J, APP/PS1 B6.Cg-Tg(APPswe,PSEN1dE9)85Dbo/J, APP8.9 B6.129S2-Tg(APP)8.9Btla/J and wild type) were housed at Rutgers University Animal Center. Brain tissues from the Tg2576 and Tg6799 mouse models were a generous gift from Dr. R. Vassar (Northwestern University). Three animals from the R1.40, Tg2576, and Tg6799 lines were examined for this study. Two each of the 8.9, APP/PS1 and wild-type strains were used.

### 2.2. Histology

Animals were anesthetized with Avertin (0.02 cc/g body weight) and perfused through the heart with 0.1 M phosphate-buffered saline (PBS), followed by 4% paraformaldehyde in 0.1 M PBS solution. The brain was immediately removed from the skull and transferred to 4% paraformaldehyde at 4°C overnight. The brains were then cryoprotected by sinking in 30% sucrose in 0.1 M PBS at 4°C overnight. After cutting along the midline, the brains were embedded in OCT compound (Tissue-Tek). Cryostat sections were cut at 10 microns and air-dried on Superfrost/Plus glass slides overnight.

For hematoxylin staining, sections were washed in PBS with 0.25% Tween-20 and rinsed in PBS. They were exposed to hematoxylin for 45–60 sec then washed with double distilled water until clear. Sections were dehydrated through graded ethanol and two washes of xylene. VectaMount was used to mount slides.

### 2.3. Immunohistochemistry

A rabbit monoclonal antibody (Abcam, Cambridge, UK) to proliferating cell nuclear antigen (PCNA) was diluted 1 : 3000 in 10% goat serum/PBS blocking buffer before use. A rabbit polyclonal cyclin A antibody (Abcam, Cambridge, UK) was used at a working dilution of 1 : 500. The beta amyloid, 1–16 (6E10) mouse monoclonal antibody (Covance, Princeton, NJ), was used at a working dilution of 1 : 3000. The anti-PHF-tau antibody clone AT8 mouse monoclonal antibody (Thermo Scientific, Rockoff, IL) was used at a working dilution of 1 : 1000.

To perform fluorescent immunohistochemistry, sections were first rinsed twice in PBS, followed by pretreatment in Antigen Unmask Solution (Vector Laboratories, Burlingame, CA; working dilution 1 : 100) for 4-5 min at 95°C. After the slides had cooled in buffer for 10–20 min at room temperature, they were rinsed twice in double distilled H_2_O. For DAB staining, slides were subjected to an additional pretreatment step: 0.3% hydrogen peroxide in double distilled water for 30 min to remove endogenous peroxidase activity. Slides were then rinsed again in double distilled H_2_O followed by PBS. Subsequently, all sections were washed with PBS and incubated for 1 h at room temperature in 10% goat serum and 0.25% Tween-20 in PBS to block nonspecific binding. All primary antibodies, diluted in PBS containing 0.25% Tween-20 and 10% goat serum, were applied to sections and then incubated overnight at 4°C. After rinsing in three washes of PBS, they were incubated for 1 h with a secondary antibody, which was conjugated with fluorescent Alexa dyes (dilution, 1 : 500). The sections were rinsed with another three washes of PBS. Antifade with DAPI was applied before sealing the sections under a glass coverslip. For DAB staining, secondary antibody (1 : 500 dilution) was applied for 1 h at room temperature, washed three times in PBS, and incubated in Vectastain ABC Elite reagent (Vector Laboratories, Burlingame, CA) for thirty minutes. After three more PBS washes, slides were developed using diaminobenzidine (DAB), following the manufacturer's protocol. Sections were dehydrated through double distilled water, graded ethanol, and washed twice in xylene. All sections were mounted in VectaMount under a glass coverslip. Control sections were subjected to the same staining procedure, except that primary antibody was omitted. Positive controls were obtained using cerebellar cortex of 15-day-old wild-type mice. 

To analyze the immunocytochemical data in a more quantitative fashion, we developed a rating scale to rate the cell cycle, AT-8, and 6E10 markers. Scores were based only for their expression in the cell body of neurons. The values assigned to the rating scale were 0 (no staining or very little staining of cell cycle events), 1 (a few staining of cell cycle events 5–15%), 2 (low staining of cell cycle events, 15–30%), 3 (moderate staining of cell cycle events 30–50%), and 4 (>50% cell cycle events). Neuronal density was examined using hematoxylin staining.

## 3. Results

### 3.1. Mouse Models

We chose to study five different mouse models. The **R1.40** YAC transgenic line contains a 650-kb yeast artificial chromosome (YAC) with the entire 400-kb *huAPP* gene modified with the Swedish mutation (K670N/M671L). R1.40 mice exhibit a preferential deposition of A*β*_1-42_, which results in the appearance of amyloid deposits in parietal cortex beginning at 13.5–14 months. In addition, the R1.40 cortex displays extensive neuritic abnormalities as evidenced by staining with APP, ubiquitin, neurofilament, and hyperphosphorylated tau antibodies [21, 22]. **Tg2576** is a commonly used AD mouse model. An APP_695_ human cDNA transgene was used, with the Swedish (K670N/M671L) double mutation, under the regulation of the hamster PrP promoter. The mice develop *β*-amyloid deposits at 9–12 months [23]. **Tg6799** is also known as the 5xTg transgenic mouse; it carries a single human APP cDNA with the Swedish K670N/M671L double mutation as well as the Florida I716V mutation, and the London V717I mutation. An additional human PSEN1 cDNA with M146L and L286V mutations was also inserted. Both cDNAs are driven by a mouse Thy-1 promoter. The mice show amyloid deposition as early as 2 months [24]. The **APP/PS1** line was generated using a mouse prion protein promoter. The transgene includes a cDNA sequence encoding the human APP gene with the Swedish mutation as well as a *PSEN1* cDNA transgene carrying the ΔE9 mutation (the sequence for exon 9 of PS1 is deleted). These were microinjected together resulting in the insertion of both *APP* and *PSEN1 *transgenes at a sinigle locus [25]. The **APP8.9** line has a YAC genomic transgene similar to R1.40, but the transgene encodes a wild-type human *APP* gene instead of the Swedish mutation found in R1.40. The levels of APP transgene expression have been found to be similar to both normal levels of *APP *expression in humans as well as to the endogenous murine *App* gene. Although no plaque pathology has been described, the APP8.9 mouse should recapitulate the *APP* dosage imbalance found in Down Syndrome [26, 27].

### 3.2. Regional Differences

**Figure 1:**
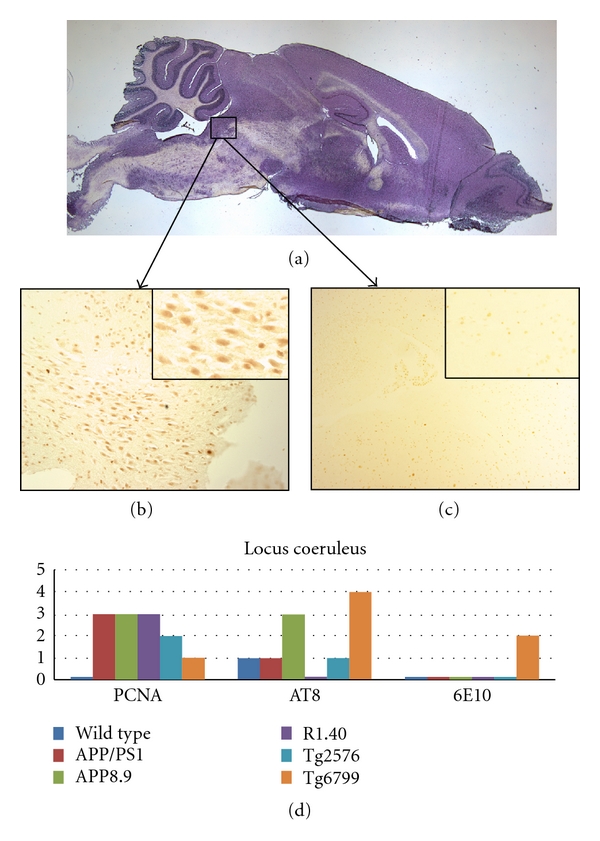
Comparison among the mouse lines studied with respect to the presence of cell cycle events (PCNA), tau-phosphorylation (AT8), and beta-amyloid plaque deposition (6E10) in the locus coeruleus. (a) Sagittal section of a wild-type mouse brain indicating the approximate location of the locus coeruleus. (b) PCNA-positive neurons are illustrated by their appearance in this representative section from the APP8.9 mouse model. (c) Minor PCNA immunostaining is seen in wild-type mice as illustrated in this representative micrograph. (d) Quantification of the extent of immunostaining for the cell cycle, phospho-tau, and beta-amyloid plaques in the five transgenic models plus wild type.

We used both DAB and fluorescent immunostaining of proliferating cell nuclear antigen (PCNA) to study the regional variations of CCEs in dorsal raphe, hippocampus, cerebellum, pons, amygdala, and locus coeruleus. PCNA is known to play an essential role in positioning the DNA polymerase in replication and repair of DNA [28]. Our results were confirmed using cyclin A, the activating subunit for several of the CDKs (cyclin-dependent kinases), which served as a second cell cycle marker. As the cyclin A results were comparable to those with PCNA, only the PCNA data are shown. The AT8 and 6E10 antibodies were used to identify neurofibrillary tangles and plaques, respectively. Immunostaining for tyrosine hydroxylase (TH) combined with their anatomical location was used to identify locus coeruleus neurons. Tryptophan hydroxylase (TpH) immunoreactivity plus anatomical location was used to identify the neurons of the dorsal raphe. The results from the locus coeruleus are illustrated in Figure 1. In 3 of the 5 models—APP/PS1, R1.40 and APP8.9 (Figure 1(b))—greater than 50% of these brainstem noradrenergic neurons were found to have immunocytochemical evidence of cell cycle activity. Tg2576 was much less affected and Tg6799 had evidence of only a few CCEs. No CCEs were detected in the wild-type locus coeruleus (Figure 1(c)). Despite the absence of cell cycle activity, Tg6799 scored the highest for phospho-tau staining, with greater than 50% of the neurons staining positive. Tg6799 was also the only model to show 6E10 staining in this region (Figure 1(d)); none of the other four AD models displayed any A*β* plaque deposition in the brainstem. A graphical summary of the CCEs and associated amyloid and tau staining patterns is shown in Figure 1(d).

**Figure 2:**
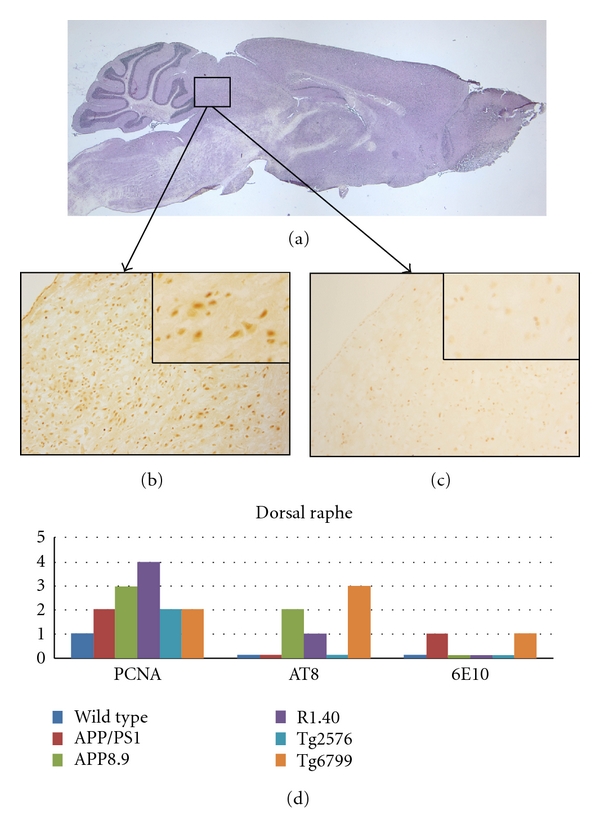
Comparison among the mouse lines studied with respect to the presence of cell cycle events (PCNA), tau-phosphorylation (AT8), and beta-amyloid plaque deposition (6E10) in the dorsal raphe. (a) Sagittal section of a wild-type mouse brain indicating the approximate location of the raphe in our preparations. (b) PCNA-positive neurons are illustrated by their appearance in this representative section from the R1.40 mouse model. (c) Minor PCNA immunostaining is seen in wild-type mice as illustrated in this representative micrograph. The insets in both (b) and (c) are representative fields at higher magnification to illustrate the qualities of the cell cycle staining. (d) Quantification of the extent of immunostaining.

The results from the dorsal raphe are shown in Figure 2. Most of the tryptophan hydroxylase immunoreactive neurons analyzed were from the region indicated by the box in Figure 2(a). Of the five models, R1.40 had the highest CCE rating (4), with greater than 50% of the neurons scoring positive for cell cycle staining (Figure 2(b)). APP8.9 had the second highest level of PCNA staining with moderate number of neurons positively stained. Age-matched wild-type mice showed virtually no cell cycle staining in this area (Figure 2(c)). Similar to our finding in the locus coeruleus, AT8 staining was variable and the dorsal raphe of the Tg6799 mouse model showed the highest levels AT8 staining. Across all genotypes, no correlation was observed between the CCE score and the presence or absence of AT8 or 6E10 immunoreactivity. 

**Figure 3:**
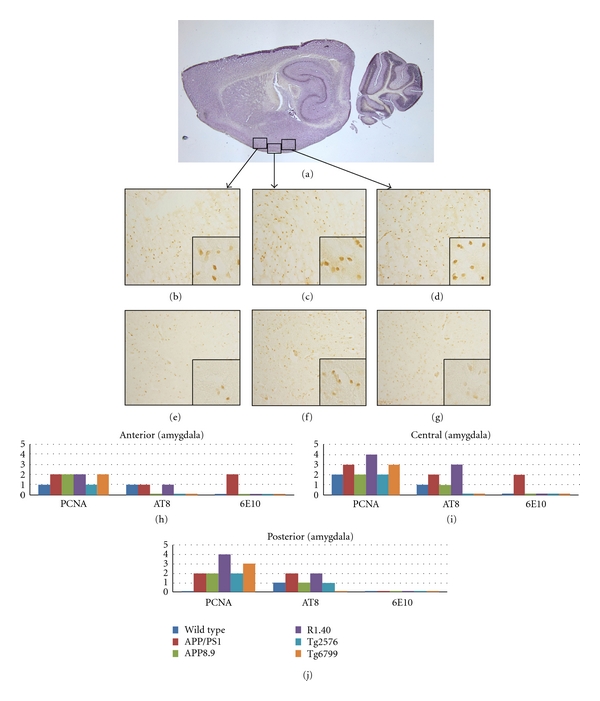
Comparison among the mouse lines studied with respect to the presence of cell cycle events (PCNA), tau-phosphorylation (AT8), and beta-amyloid plaque deposition (6E10) in the amygdala. (a) Sagittal section of a wild-type mouse brain indicating the approximate location of the amygdala. The three black boxes indicate the regions identified as anterior (left), middle (center) and posterior (right). (b)–(g) Representative fields illustrating the involvement of cell cycle processes in the three regions. The insets are representative fields shown at higher magnification to illustrate the qualities of the cell cycle staining. Anterior amygdala for R1.40 (b) and wild-type (e) mice contains the lowest density of CCEs. Middle regions of the amygdala in R1.40 (c) and wild-type (f) mice show increased staining in most models. Posterior amygdala in R1.40 (d) but not wild type (g) animals also show cell cycle activity. (h) Quantification of the extent of immunostaining for the cell cycle, phospho-tau, and beta-amyloid plaques in the five transgenic models plus wild type.

Our analysis of the amygdala was performed in sagittal sections, which makes the reliable identification of the specific subnuclei more difficult. To address this we divided the structure into three subregions as illustrated by the black boxes in Figure 3(a). The anterior region contained predominantly the anterior cortical amygdaloid nucleus, with small contributions of the nuclear ansae lenticularis and the medial and cortical amygdaloid nuclei; the middle region contained predominantly the posterolateral cortical, with lesser amounts of the anterior-lateral and central amygdaloid nuclei; the posterior region contained predominantly the posteromedial cortical amygdaloid nucleus with small contributions from the medial basal amygdaloid nucleus. Using this scheme, regional differences were found in the involvement of neuronal cell cycle events in the AD models. For all five models, the anterior portion of the amygdala demonstrated lower levels of cell cycle staining, while the medial and posterior portions showed higher levels of staining. Of the 5 transgenic models, R1.40 showed the highest level of immunoreactivity, with greater than 50% of the neurons in the posterior and medial portions of the amygdala staining positive for cell cycle events. Tg6799 had a slightly lower CCE score, followed by APP/PS1. Surprisingly, the wild-type mice showed modest levels of CCEs in the anterior and medial portions, but no CCEs in the posterior portions. Tg2576 and APP8.9 showed little staining or no staining in any of the three regions. In two models, R1.40 and APP/PS1, AT8 was distinctively higher in the posterior and medial regions. Other models had lower levels of AT8. Amyloid deposits in the amygdala, as revealed by 6E10 staining, were observed only in the anterior and medial regions of APP/PS1 mice. 

All other regions of the CNS were substantially negative for cell cycle protein expression, as expected. The one exception to this was a small region of the ventral brainstem. In the pons and the more dorsal nucleus reticulari tegmentis pontis (NRTP), significant cell cycle protein expression was identified for all animals. Wild-type animals showed moderate levels of CCEs, with similar levels in the APP8.9 and Tg2576 APP/PS1 and Tg6799. The only exception to this pattern was found in the R1.40 model, which had low levels of CCEs in this region. The R1.40 showed high pontine levels of hyperphosphorylated tau, but low levels of 6E10-positive beta amyloid deposits. Tg6799 exhibited a very high level of 6E10 staining—at greater than 50%, the highest score of any of the models. 

**Figure 4:**
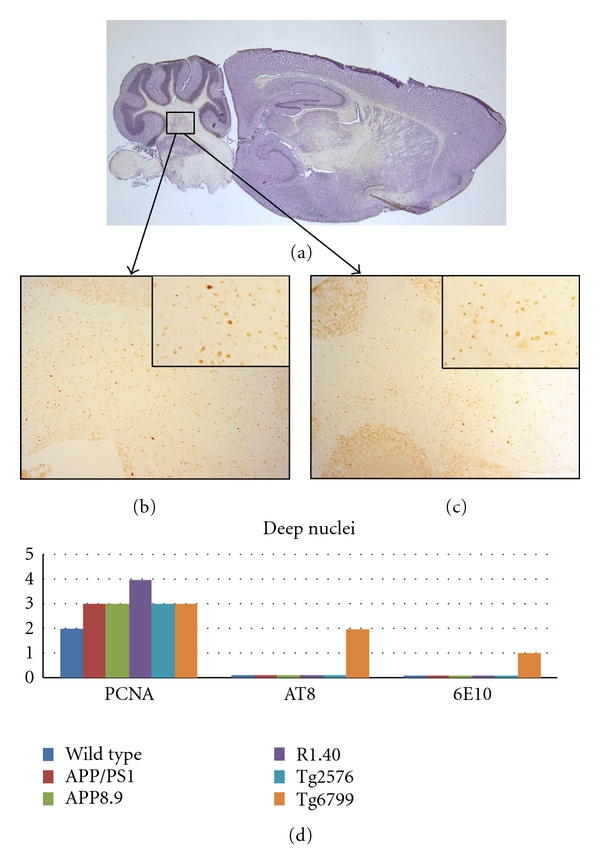
Comparison among the mouse lines studied with respect to the presence of cell cycle events (PCNA), tau-phosphorylation (AT8), and beta-amyloid plaque deposition (6E10) in the deep cerebellar nuclei. (a) Sagittal section of a wild-type mouse brain indicating the approximate location of the deep nuclei. (b) PCNA-positive neurons are illustrated by their appearance in this representative section from the Tg2576 mouse model. (c) Curiously, some PCNA immunostaining is also seen in wild-type mice as illustrated in this representative micrograph. The insets in both (b) and (c) are representative fields shown at higher magnification to illustrate the qualities of the cell cycle staining. (d) Quantification of the extent of immunostaining for the cell cycle, phospho-tau, and beta-amyloid plaques in the five transgenic models plus wild type.

In addition to these results, there were observations of cell cycle staining that were more unexpected. For example, the deep nuclei of the cerebellum showed strong levels of CCEs in almost all the models (Figure 4). While AT8 and 6E10 staining was observed only in Tg6799 mice, four out of five transgenic models showed moderate CCE staining; R1.40 showed greater than 50% of the neurons positive for CCE staining. A more detailed analysis of the involvement of the cerebellar deep nuclei in the pathogenesis of AD is the subject of a newly published report [29]. 

**Figure 5:**
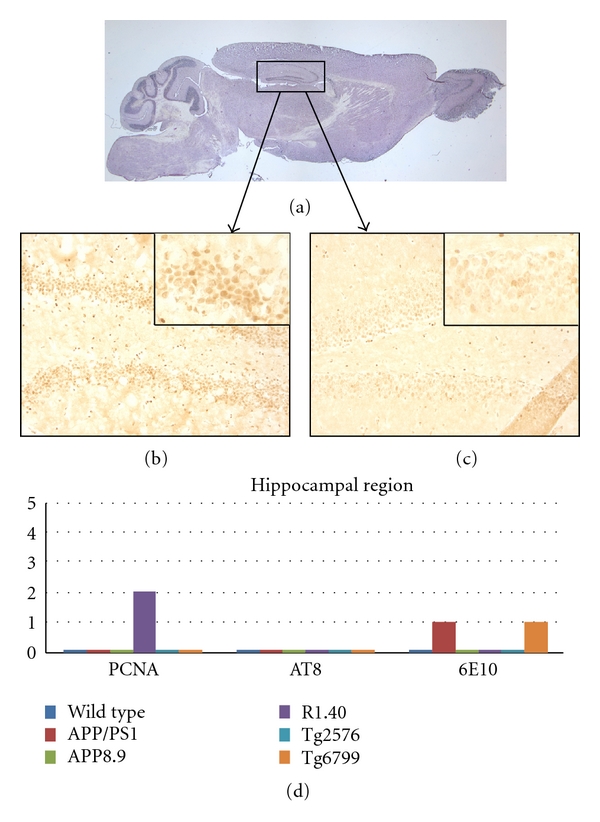
Comparison among the mouse lines studied with respect to the presence of cell cycle events (PCNA), tau-phosphorylation (AT8), and beta-amyloid plaque deposition (6E10) in the hippocampus. (a) Sagittal section of a wild-type mouse brain indicating the approximate location of the areas illustrated in (b) and (c). (b) PCNA-positive neurons are illustrated by their appearance in this representative section from the R1.40 mouse model. Note the involvement of a subset of the dentate granule cells as well as a few CA4 pyramidal neurons (arrows). (c) Minor PCNA immunostaining is seen in wild-type mice. The insets in panels (b) and (c) represent higher magnification of the CA2 region of their respective mouse model. (d) Quantification of the extent of immunostaining for the cell cycle, phospho-tau, and beta-amyloid plaques in the five transgenic models plus wild type. Note that only the R1.40 model showed cell cycle protein expression at the ages we examined.

The most unexpected finding, however, was in the hippocampal formation. CCEs have been reported in this region for two mouse models of AD, the PDAPP mouse and the R1.40 model [18]. In the current study, we did not choose to explore the PDAPP mice, but were able to replicate our findings in the R1.40 (Figure 5(b)). Both dentate granule neurons and pyramidal cells show cell cycle activity. Curiously, none of the other models we examined had evidence of cell cycle activity in this region. We observed no significant AT8 or 6E10 staining in the dentate gyrus or in the pyramidal cells of any of the mouse models (Figure 5(d)).

## 4. Discussion

Transgenic mouse models have long been used to study the molecular mechanisms of disease. To be considered useful, such models must recapitulate the human disease in as many ways as possible, and by this criterion the mouse models of AD have been at least partially successful. No wild-type mouse has been reported to naturally develop amyloid plaques, neurofibrillary tangles, or an Alzheimer's-like loss of neurons in any brain region. By contrast, mouse lines expressing *APP* and/or *PSEN1* transgenes with AD-related mutations display an age-related appearance of *β*-amyloid plaques and hyperphosphorylated tau [30]. Behavioral and neurophysiological abnormalities have also been observed in some transgenic models, as well as neurophysiological defects, inflammation, and occasionally a decrease in the numbers of CA1 pyramidal neurons [31]. Impressively, the YAC R.140 model shows a regional pattern of cerebral and vascular amyloid deposits along with reactive astrocytes and microglia that is consistent with the pattern of these events in human AD. In the end, however, the reproduction of the human disease has been incomplete in all of these lines. No neurofibrillary tangles are found; neurodegeneration is limited even in the best models; and the behavioral changes are mild compared to those observed in humans with mid- to late-stage Alzheimer's disease. Also, because most successful *APP*-expressing mouse lines require highly elevated levels of transgene expression to form plaques, the use of plaque deposition as the major outcome measure for evaluating the effectiveness of the models represents somewhat of a self-fulfilling prophecy [32]. This raises concerns that the effectiveness of the current *APP-*based AD mouse models might be compromised.

One of these concerns that we attempt to address here is the poor reproduction in the mouse of the neuronal cell death found in AD. In human AD, there is a massive degeneration of neurons and this is observed in a pattern with pronounced temporal and regional variability [33]. While the reasons for the discordance between mouse and human phenotypes are unknown, if CCEs are used in place of actual neuronal cell loss as an index of neurodegeneration, our previous studies of the R1.40 model suggest a remarkably faithful replication of the progression of neuronal cell death in human AD. What remains unknown is why, if CCEs are correlated with neuronal cell death, the subsequent death of neurons is not immediate. Indeed, studies in both mouse and human suggest that cell cycling and cell death can be months apart [19, 34]. 

To the appearance of cell cycle events, we add the correlation with more traditional neuropathological indicators of Alzheimer's disease. Our findings show that *β*-amyloid deposits appear in several subcortical regions—not just in the R1.40 mouse [34], but in most models. The anatomical distribution of the deposits, however, is not identical. *β*-amyloid deposition appears in some but not all of the structures studied and some but not all of the models stain with the AT8 phospho-tau antibody. Based on the models we examined, it would appear that amyloid deposits and tau expression levels showed region- and model-specificity and thus cautions should apply. 

In this study, we have expanded the range of biological responses in the 5 different AD mouse models to include cell cycle events (CCEs) as direct cell-autonomous indices of neuronal distress. Since CCEs have been observed in both the human AD brain and in the analogous regions of certain AD mouse models, characterizing CCE expression patterns provides a logical and independent outcome measure for the study of neuronal death process in human AD. It is significant, therefore, that the results reported here demonstrate clear differences among the mouse models examined in the pattern of CCE expression. We find a high level of CCEs in the brain stem of all 5 models, including the pons, locus coeruleus, dorsal raphe, and deep nuclei of the cerebellum. The consistent appearance of CCEs in these more caudal regions of the CNS despite differing transgene properties and *β*-amyloid and tau pathologies is significant. It implies that the existence of neuronal stress in metencephalic and myelencephalic regions in familial AD may represent a central feature of disease pathogenesis. This is consistent with previous suggestions arising from entirely different lines of evidence. Immunocytochemical and biochemical techniques revealed that cells produced from a locus coeruleus-derived cell line, but not hippocampal and cortical neurons, exhibited beta-amyloid accumulation and concentrate *β*-secretase at process terminals [35]. In the same study, it was shown that intracellular A*β* plaques can become extracellular when neurites degenerate, which leads to additional accumulation and extracellular aggregation. In a different domain, the locus coeruleus has been proposed to play a role in brain inflammatory processes, such as those seen in AD [36, 37]. More recently, Braak and Tredici, using tau phosphorylation as an index, also report very early disease pathology in the locus coeruleus [38].

The pattern of CCEs in the amygdala suggests internal variations in AD pathology in this structure such as is seen in the cortex. In several AD models, the posterior and central amygdaloid nuclei showed stronger levels of CCE expression when compared to more anterior amygdaloid neurons, suggesting a common impact of APP transgene expression in this region. This variation is not apparent in the 6E10 or AT8 staining suggesting that specific neuroanatomical phenotypes may be uncovered when CCE markers are used. Our findings in the AD mouse models are consistent with the well-documented involvement of the structure in human AD pathology [39–41]. These studies have shown that significant numbers of neurons in the amygdala die during the early stages of AD. Although there is less information available on the regional variability within the amygdala, attempts have been made to use degeneration in this structure to detect the onset of AD [34, 42]. 

The R1.40 model showed the strongest staining for CCEs in most subcortical regions and was the only model in the current study to show significant CCE expression in the hippocampal region. The absence of CCE expression in the 4 other AD models we studied is noteworthy, albeit without explanation. Nearly all of the models we studied have been shown to have deficits in behavioral tasks that are known to involve the hippocampus. This discordance between function and pyramidal cell body neuropathology suggests several hypotheses, none of which are mutually exclusive. Perhaps the behavioral changes are due to synaptic loss or atrophy [43], but the cell body, as seen through the appearance of CCEs or neuronal cell death, remains largely unaffected. A related hypothesis is that the transgene-dependent excess of A*β* at the synapse is the cause of the behavioral and physiological changes. A*β*, especially the lower molecular weight form, is recognized as having a neuromodulatory function [44]. Finally, as the ages of the animals we examined were mostly one year or less, it is also possible that the disease process in the hippocampus was not sufficiently advanced at the time of perfusion. 

All of these alternatives are consistent with the proposal that Alzheimer's begins as a synaptic disease. What is unknown at present is whether these synaptic problems precede the CCEs. This would appear to be the situation in hippocampus where slices, isolated in vitro, show impaired LTP [45]; whether this accounts for all of the behavioral changes or whether some might be due to the aberrant cell cycle activity is unknown at this time.

Although no single mouse model provides a complete recapitulation of human AD, based on the regions we examined, the YAC R1.40 would appear to be the most reliable model, especially when using CCEs as an outcome measure. One possible explanation for R1.40's strong fidelity as a model is the close reproduction of the pattern of transgenic *APP* expression to that found in human. This in turn is most likely due to the method used to insert the mutant gene [21, 22]. With the exception of APP8.9, the other transgenes encode a single splice form of the human *APP *cDNA. The R1.40 mouse model carries the entire human *APP *gene, including all introns and all 3′ and 5′ regulatory elements within 30–50 kb of the coding sequence. This allows for a more faithful temporal and spatial expression pattern, possibly contributing to a more faithful reproduction of the human disease.

## 5. Conclusions

We have shown here that CCE markers are a reasonable way of studying AD mouse model fidelity to human AD. Since no transgenic mouse model is able to perfectly capture the complexities of the human AD pathology, using several phenotypic markers to study the effects of transgene insertions is well advised. Distinguishing the role of species differences and the effects of transgenes in AD pathogenesis through rigorous characterization of mouse models and AD will be important to uncovering the mechanisms of AD pathogenesis and lead to the more rapid identification of useful therapeutic targets.

## Figures and Tables

**Table 1:** Transgenic mouse lines used in this study.

Transgenic lines	Strain	Approach	Mutation	Promoter	A*β* deposits (age of onset)	Age of analysis (this study)
R1.40	B6.129-Tg (APPSw) 40Btla/J	YAC Genomic	Swedish APP	Human APP	14 months	1 yr; 2 yrs
Tg2576	B6 SJL-Tg (APPsw)	cDNA (695)	Swedish APP	Hamster PrP	9–12 months	9–11 months
Tg6799 (5xTg)	B6SJL-Tg (APPSwFlLon, PSEN1*M146L*L286V) 6799Vas/J	Pronuclear coinjection : APP and PS1 transgenes	Swedish, Florida and London APP & human PS1	Murine Thy-1	3 months	6 months
APP8.9	B6.129S2-Tg (APP) 8.9Btla/J	YAC Genomic	Wt huAPP	Human APP	N/A; same as mouse APP expression	14 months
APP/PS1	B6.CgTg (APPswe, PSEN1dE9) 85Dbo/J	cDNA	Swedish/human PS1	Murine PrP	6 months	6-7 months
